# MicroRNA319-mediated gene regulatory network impacts leaf development and morphogenesis in poplar

**DOI:** 10.48130/FR-2021-0004

**Published:** 2021-02-05

**Authors:** Yanxia Cheng, Lihu Wang, Manzar Abbas, Xiong Huang, Qiao Wang, Aimin Wu, Hairong Wei, Shaobing Peng, Xinren Dai, Quanzi Li

**Affiliations:** 1 College of Forestry, Northwest A & F University, Yangling, Shaanxi 712100, P.R. China; 2 State Key Laboratory of Tree Genetics and Breeding, Chinese Academy of Forestry, Beijing 100091, P.R. China; 3 College of Landscape and Ecological Engineering, Hebei University of Engineering, Handan, Hebei 056038, P.R. China; 4 Key Laboratory of Aromatic Plant Resources Exploitation and Utilization, School of Agriculture, Forestry and Food Engineering, Yibin University, Yibin, Sichuan 644000, P.R. China; 5 Research Institute of Forestry, Chinese Academy of Forestry, Beijing 100091, P.R. China; 6 State Key Laboratory for Conservation and Utilization of Subtropical Agro-bioresources, South China Agricultural University, Guangzhou, Guangdong 510642, P.R. China; 7 Guangdong Key Laboratory for Innovative Development and Utilization of Forest Plant Germplasm, College of Forestry and Landscape Architecture, South China Agricultural University, Guangzhou, Guangdong 510642, P.R. China; 8 College of Forest Resources and Environmental Science, Michigan Technological University, Houghton, Michigan 49931, USA

**Keywords:** poplar, miR319a, leaves, TCP transcription factors, transgenic plants

## Abstract

MicroRNA319 (miR319) has been implicated in leaf development in a number of plant species. Here we study the roles of miR319a and its regulated network in leaf development in poplars. Over-expression of miR319a in *Populus alba* × *Populus glandulosa* caused dwarf statures, narrow leaf blades and serrated leaf margins. The vascular bundles and bundle sheaths in transgenic leaves had more layers of cells than those in the leaves of control plants, indicating enhanced lignification in these cells. Among the 93 putative targets of miR319a predicted with the psRNATarget tool, only three genes, *TCP* (*TEOSINTE BRANCHED1*, *CYCLOIDEA*, and *PROLIFERATING CELL NUCLEAR ANTIGEN BINDING FACTOR*), were differentially expressed in the leaves of *MIR319a*-over-expression transgenic lines. With the RNA-seq data sets from multiple *MIR319a* over-expression transgenic lines, we built a three-layered gene regulatory network mediated by miR319a using Top-down graphic Gaussian model (GGM) algorithm that is capable of capturing causal relationships from transcriptomic data. The results support that *miR319a* primarily regulates the lignin biosynthesis, leaf development and differentiation as well as photosynthesis via *miR319-MEE35/TCP4*, *miR319-TCP2* and *miR319-TCP2-1* regulatory modules.

## INTRODUCTION

MicroRNAs (miRNAs) are small (18-25 nucleotides) endogenous RNAs that regulate many biological processes like growth, nutrient homeostasis, hormone signalling, stress response and metabolism by post-transcriptional silencing and chromatin regulation^[1-4]^. For example, modulation of many cellular processes and regulation ensues when some transcriptional products, especially mRNAs and transcriptomic profiles, are targeted by miRNAs for degradation^[5]^. When some miRNAs and their target transcription factors (TFs)^[6-9]^ form various regulatory modules, for instance, TF-miRNA-mRNA and miRNA-TF-mRNA, they can exert powerful, delicate inhibitory/stimulatory regulation on evolutionarily conserved functions, such as organogenesis and senescence among a broad range of species^[2]^.

Based on the miRBase Registry 22.116.0 (http://www.mirbase.org), there are currently 587 curated mature miRNAs that play important regulatory roles in the growth and development of *Populus trichocarpa*^[10]^. Some conserved developmental processes in plants, that are known to be governed by miRNAs, include: leaf morphogenesis, flower development, transition from vegetative growth to reproductive growth, and senescence stage^[11-13]^. Up-regulation of miR393 in *Malus domestica* undermined fungal pathogenicity^[14]^. miR156 and miR167 are dominantly expressed in leaves and floral buds to regulate fruiting in *Malus domestica*^[14]^. Identification and *in silico* analysis of miRNAs in *Camellia sinensis*^[15]^ revealed the roles of miR164 and miR169 in leaf primordia and root development respectively. Transcriptomic analysis unravelled that miR950 and miR1309 are dominantly expressed in young needles of *Pinus contora* to regulate chloroplast-specific genes^[16]^. miR1310 and miR1314 are gymnosperm-specific and differentially expressed under methyl jasmonate application in *Taxus chinensis*; miR1310 reduces oxidative damage while miR1314 regulates cellulose synthase genes^[17]^.

miR319 belongs to one of the most ancient and conserved miRNA families^[18-20]^ and is conserved in both angiosperms and gymnosperms^[21]^. In *Arabidopsis thaliana*, the miR319 family is composed of seven genes (*MIR319a-g*)^[22]^. It has been shown that miR319-targeted transcription factors, *TEOSINTE BRANCHED* / *CYCLOIDEA* / *PROLIFERATING CELL FACTORS* (*TCP*) genes^[12,13,23]^ play important roles in plant development such as regulating cell proliferation in leaf morphogenesis^[11,13,24,25]^. Overexpression of miR319 in *A. thaliana*^[6]^, tomato^[24]^ and rice^[26]^ resulted in continuous leaf marginal growth, altered leaf curvature and delayed flowering, while over-expression of the switchgrass *Pvi-MIR319a* precursor gene in rice, gave rise to broader leaves and delayed flowering than of that in the control^[27]^. Overexpression of rice Osa-miR319a in creeping bentgrass also caused significantly greater leaf expansion (blade width and vein number) and thicker leaves^[23]^. In switchgrass, over-expression of rice Osa-miR319a also showed significantly wider leaves and narrower leaf blades^[25]^. A moderate pause in leaf serrations was observed in *A. thaliana* with a single mutation in miR319a and miR319b in *Arabidopsis*^[9,28]^. In rice, over-expression of Osa-miR319b represses the expression of *OsPCF6* and *OsTCP21* and results in enhanced tolerance to cold stress partially through modifying active oxygen scavenging^[29]^. In poplar, over-expression of miR319 suppresses TCP4, which, in turn, activates VND7^[30]^, a high hierarchical regulator regulating secondary wall formation^[31]^. In addition, miR319 controls TCP4 that activates LIPOXYGENASE2 functioning in conversion of α-linolenic acid (18:3) into (13S)-hydroperoxyoctadecatrienoic acid, the first dedicated step in the biosynthesis of oxylipin jasmonic acids^[12]^. In brief, the roles of miR319 and its target genes form different regulatory modules that are primarily involved in the regulation of leaf development, secondary wall formation, and secondarily in stress responses and hormone biosynthesis^[23,26]^.

In this study, we investigated the roles of miR319 in poplar leaf development by over-expression of miR319a in *P. alba* × *P. glandulosa*. We identified 93 potential target genes and then employed the top-down GGM algorithm^[32,33]^, to build a three-layered gene regulatory network mediated by miR319a. The network showed that miR319a directly regulated three *TCP* genes, which in turn controlled the genes involved in hormone synthesis/transport, photosynthesis and growth.

## RESULTS

### *MIR319a* overexpression affected leaf development in poplar

**Figure 1:**
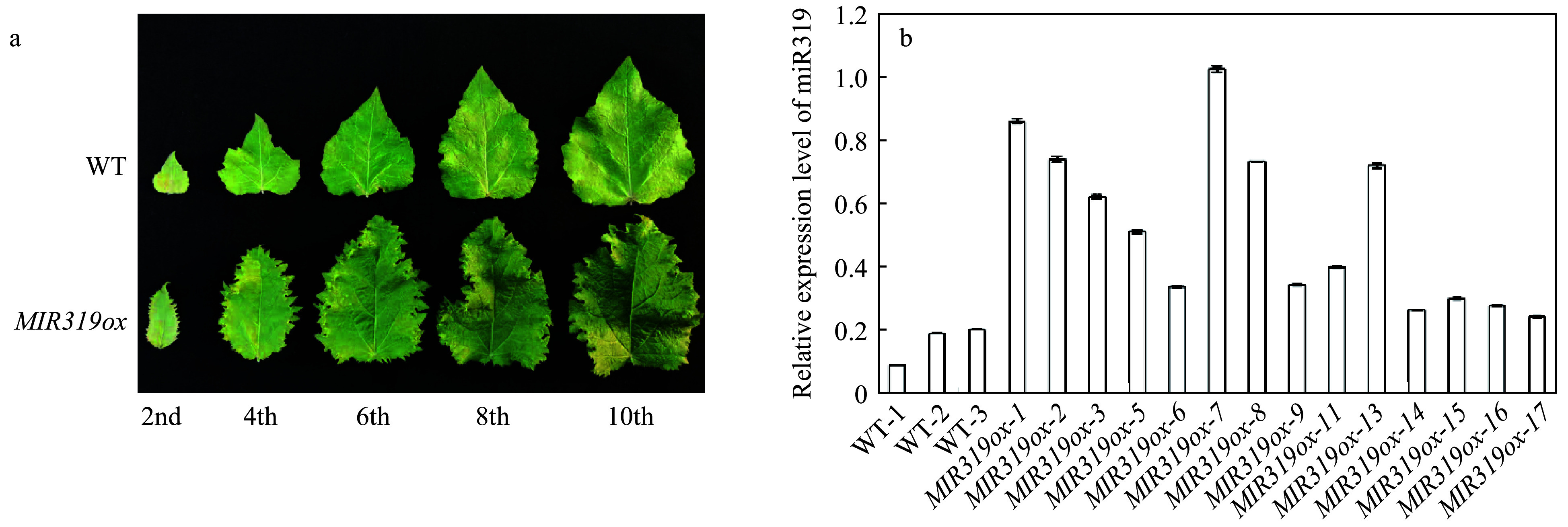
Phenotypic characterizations of *MIR319a* over-expression transgenic polar lines. (a) Leaves from *MIR319a* over-expression transgenic lines and WT. The 2nd, 4th, 6th, 8th, and 10th represent the second, fourth, sixth, eighth and tenth leaves counted from the apical bud of *MIR319a* over-expression transgenic poplar plants, respectively. (b) The expression levels of miR319a in WT and 14 transgenic lines measured by qRT-PCR with three technical replicates.

To study the functions of miR319a in poplar, we obtained plasmid *p35S-Osa-miR319a/p35S-hyg*^[23,34]^ that harbours a CaMV 35S promoter-driven *Osa-MIR319a* gene from *Oryza sativa*, and transformed *P. alba* × *P. glandulosa* via *Agrobacterium* to obtain *MIR319a* over-expression transgenic lines. A total of 17 transgenic lines, *MIR319ox-1* to *MIR319ox-17*, were obtained and grown in a plant growth room, but three of them, *MIR319ox-4*, *-10*, and *-12*, failed to survive. All transgenic lines manifested different phenotypes on leaf shapes as compared to the wild-type (Fig. 1a). Most obviously, the top leaves of *MIR319a* transgenic plants were longer and slightly whiter in color than those of the wild-type. The mature leaves were thicker and curlier. More obviously, the leaves showed irregular jagged leaf edges, which were absent in the leaves of non-transgenic poplar.

In contrast, the wild type plants did not change dramatically in leaf color, shape and margins as the plants aged (Fig. 1a). *MiR319a* over-expression transgenic plants showed largely similar leaf phenotypes as those in *A. thaliana*^[11]^, tomato^[24]^, and rice^[26]^, suggesting a conserved function of miR319a in controlling leaf development. We used qRT-PCR^[35]^ to verify the expression of miR319a in the leaves of 14 transgenic lines. The expression levels of miR319a in transgenic leaves were significantly higher than those in WT (Fig. 1b). Among all 14 transgenic lines, *MIR319ox-7*, which had the highest expression level of miR319a (Fig. 1b), manifested the most severe phenotype alteration. The degrees of leaf curling and jaggedness appeared to be aggravated as the expression level of miR319a increased (Fig. 1b and Supplemental Fig. 1).

**Figure 2:**
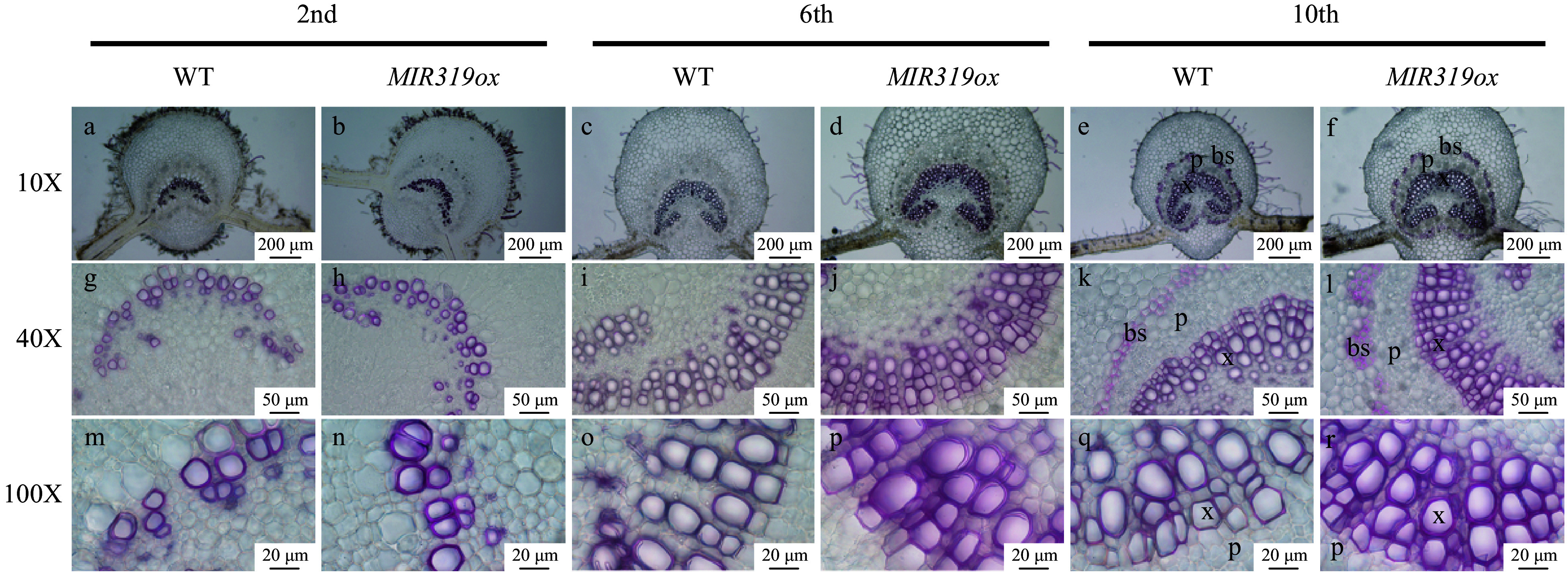
The vascular bundle and bundle sheath cells on microscopic leaf cross sections stained with phloroglucinol HCl in *MIR319a* over-expression transgenic lines and WT. The leaves were from the second, sixth and tenth stem nodes, respectively. The photos were taken at 10X, 40X and 100X. Bars in a−f: 200 μm, g−l: 50 μm, m−r: 20 μm. x, p and bs represent xylem, phloem and bundle sheath, respectively.

To investigate the effects of miR319a over-expression on leaf anatomical structures, we prepared microscopic cross-sections of the main veins of transgenic leaves from second, sixth and tenth nodes in the line *MIR319ox-7*, which had the highest expression level. Phloroglucinol hydrochloride was then used to stain the main veins of leaves in these cross-sections to manifest lignin. We observed a significantly increased number of stained cells in the vascular bundle and more layers of cells in vascular bundle sheaths in the leaves of the *MIR319a* over-expression transgenic line (Fig. 2), suggesting that over-expression of miR319a enhanced lignification of the cells in vascular bundles and vascular bundle sheaths of leaves.

### Analysis of RNA-seq data to identify differentially expressed genes (DEGs)

To study the effect of miR319a overexpression on gene expression in leaves, we performed high-throughput RNA-seq for the leaves from 14 *MIR319a* over-expression transgenic lines and three WT plants. We used the five transgenic lines (*MIR319ox-1*, *-2*, *-7*, *-8* and *-13*) with the highest miR319a expression levels as the treatment group and WT as the control group. 1342 DEGs were identified (Supplemental Table 1), of which 543 were up-regulated and 799 were down-regulated.

### Genome-wide identification of putative targets of miR319a in poplar

We deployed a psRNATarget^[36]^ tool to identify the target genes of miR319a in the *P. trichocarpa* genome using a threshold expectation value equal to and less than 5. A total of 93 putative target genes were identified (Supplemental Table 2). Among these 93 putative target genes, a few *TCP* genes including Potri.012G109000, Potri.004G065800, Potri.011G083100, Potri.011G096600, and Potri.013G119400, which had small expectation values, emerged at the top of the list. Only three of 93 target genes were among the 1342 DEGs identified from *MIR319a* over-expression lines. These three genes were *MEE35/TCP4* (Potri.001G375800), *TCP2* (Potri.004G065800) and *TCP2-1* (Potri.011G083100), showing that the number of potential target genes whose expression levels were significantly modulated by miR319a in leaves. Owing to similarity in sequences, Potri.001G375800 may have similar functions with *Arabidopsis* *MEE35/TCP4* in that it participates in the heterochronic regulation of leaf differentiation (Supplemental Table 2). Auxin, gibberellic acid and abscisic acid have been thought to participate in miR319-TCPs-mediated control of leaf growth^[37,38]^. In our putative target genes, some function in the roles of phytohormone biosynthesis or signaling pathways. For example, *ASA1* (*Potri.017G101100*) encodes the alpha subunit of anthranilate synthase, which catalyzes the rate-limiting step of tryptophan (Trp) synthesis. Trp is a precursor for the auxin biosynthesis pathway^[39]^; *Potri.001G036000* and *Potri.001G224500*, both encode a MYB65 protein, are the ortholog of HvGAMYB that is inducible by GA during germination in barley^[40,41]^.

**Figure 3:**
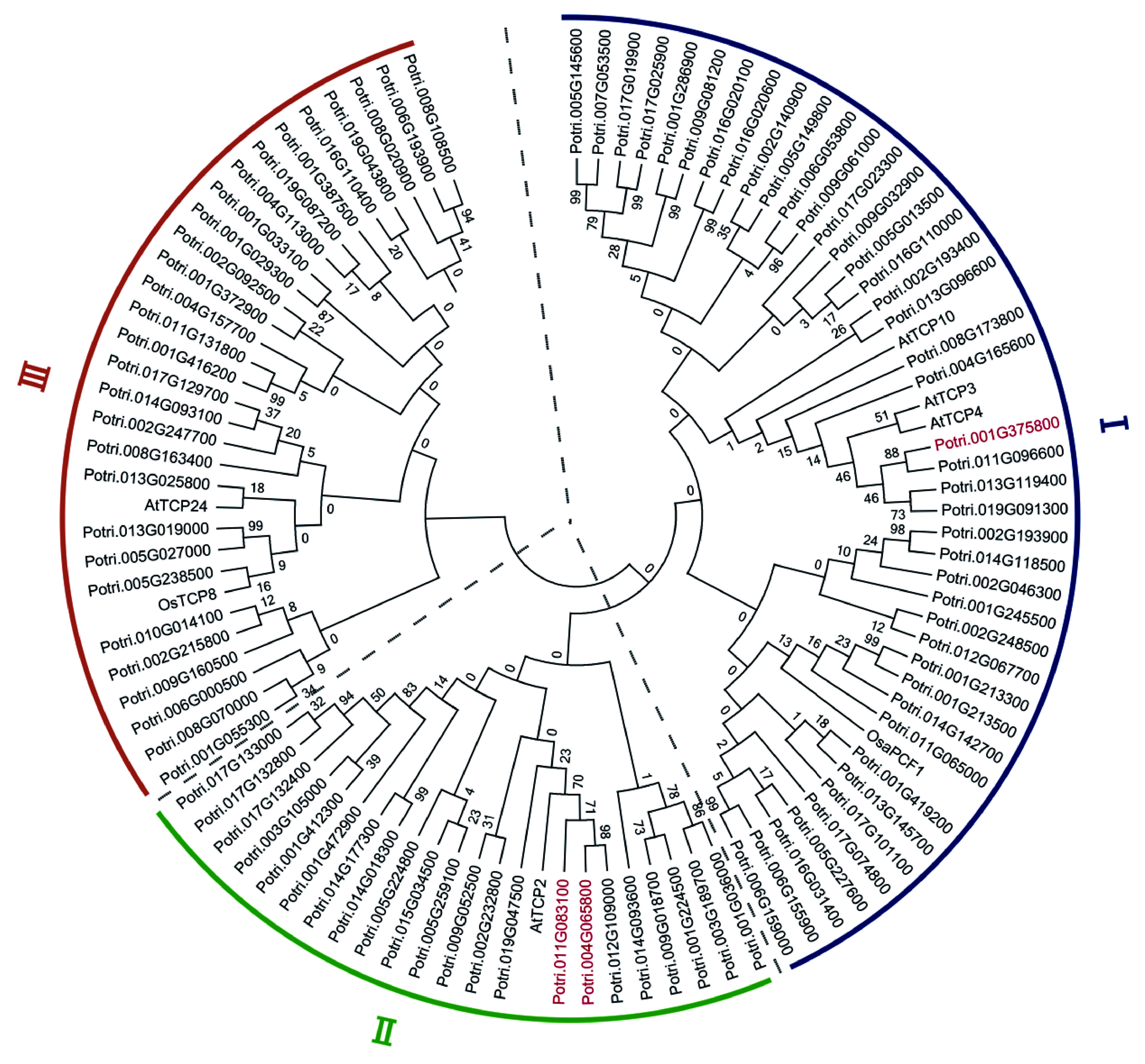
Phylogenetic tree constructed with the protein sequences of 93 putative target genes of miR319a in *Populus trichocarpa*, 5 from *Arabidopsis* and 2 from rice. A neighbor-joining (NJ) method in MEGA7.0 was used to generate the tree. The tree consists of 3 distinct clades. The proteins whose genes were differentially expressed genes (DEGs) are highlighted in red.

To study evolutionary relationships of the miR319a-regulated TCPs in comparison with those in *Arabidopsis* and rice, we constructed an unrooted phylogenetic tree using MEGA 7.0 with all TCP protein sequences from *P. trichocarpa,* and some from *A. thaliana* and rice. A total of 42 TCPs were clustered in the first clade along with AtTCP3, AtTCP4, AtTCP10 and OsaPCF1, whereas 22 clustered in the second clade along with AtTCP2, and 29 grouped in the third clade along with AtTCP24 and OsTCP8 (Fig. 3). For the three direct target genes of miR319a, MEE35/TCP4 presented in the first clade, while TCP2 and TCP2-1 appeared in the second clade together with AtTCP2 that is a direct target of miR319a in *Arabidopsis*^[42]^.

Phylogenetic tree constructed with the protein sequences of 93 putative target genes of miR319a in *Populus trichocarpa*, 5 from *Arabidopsis* and 2 from rice. A neighbor-joining (NJ) method in MEGA7.0 were used to generate the tree. The tree consists of three distinct clades. The proteins whose genes were differentially expressed genes (DEGs) are highlighted in red.

### Determination of miR319a direct target gene expression

**Figure 4:**
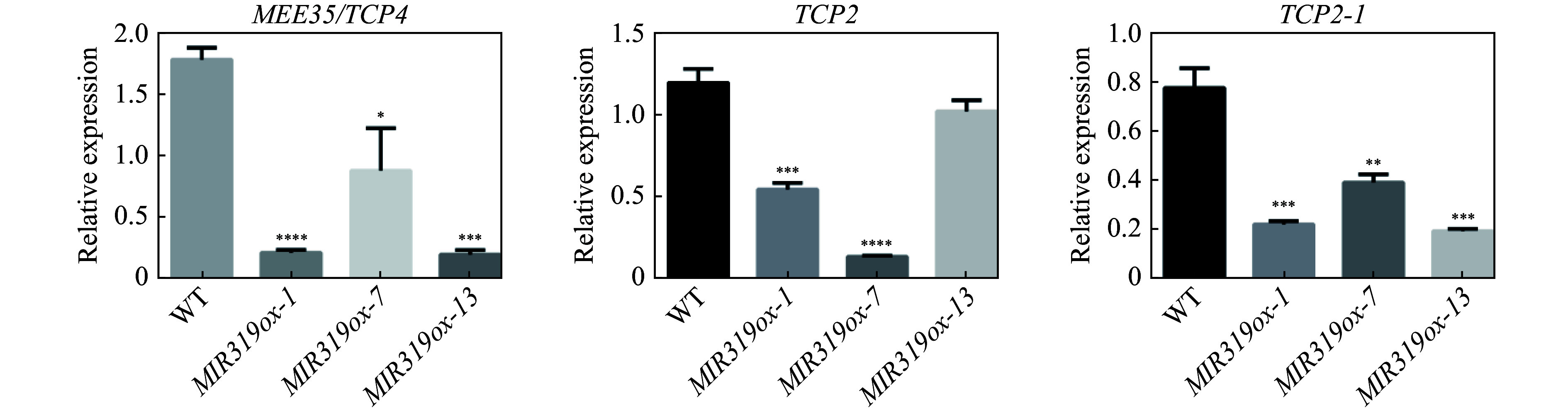
Expression levels of *MEE35*/*TCP4*, *TCP2* and *TCP2-1* determined by qRT-PCR in *MIR319a* over-expression transgenic leaves and WT.

The three direct target genes (*MEE35/TCP4*, *TCP2* and *TCP2-1*) of miR319a, which were identified by intersecting the target genes identified by psRNATarget and the DEGs in *MIR319a* transgenic lines, were examined by qRT-PCR for their expression levels. *MIR319ox-1*, *MIR319ox-7* and *MIR319ox-13*, the three *MIR319a* over-expression transgenic lines with the highest expression levels of miR319a, were chosen to examine the expression levels of *MEE35/TCP4*, *TCP2* and *TCP2-1* genes. The results showed that *MEE35/TCP4*, *TCP2* and *TCP2-1* were significantly down-regulated compared to those in the WT plants (Fig. 4). For example, the transcript abundances of *TCP2*, *TCP2-1* and *MEE35/TCP4* in *MIR319ox-1* were down-regulated by 54.0%, 71.0%, and 87.6%, respectively. In *MIR319ox-7*, the percentages of the expression levels of *TCP2*, *TCP2-1* and *MEE35/TCP4* reduced 88.2%, 49.2%, and 49.9%, respectively. These results show that over-expression of miR319a significantly down-regulated *MEE35/TCP4*, *TCP2* and *TCP2-1* in transgenic *P. alba* × *P. glandulosa* leaves.

### Gene ontology (GO) and domain enrichment analysis

To study which pathway and biological processes are affected by miR319a over-expression, the gene ontology (GO) enrichment and protein domain enrichment were analyzed with Pop’s pipe^[43]^ using the 1342 DEGs as an input. Gene ontologies that include 222 biological processes (GO-type P), 33 cellular components (GO-type C) and 55 molecular functions (GO-type F) were significantly over-represented by these DEGs (Supplemental Table 3). We further underscored nine significantly enriched biological processes: chlorophyll biosynthetic and metabolic process, photosynthesis, leaf and phyllome development, meristem development and maintenance, regulation of meristem development, xylem histogenesis and development, lignin metabolic process, tryptophan catabolic process, and gibberellin biosynthetic process (Table 1 and Supplemental Table 4). The analysis indicated that the photosynthesis and some linked biological processes, phyllome development, and lignin catabolic processes were generally down-regulated, whereas leaf development, meristem development and maintenance, phloem and xylem histogenesis and development, lignin metabolic process, and hormone synthesis and metabolism were largely augmented in the *MIR319a* over-expression transgenic leaves, which are consistent with the results of previous studies on miR319^[37,38,44,45]^.

**Table 1:** Gene ontology enrichment analysis of differentially expressed genes (DEGs) in *MIR319a* over-expression transgenics.

GO_Term	GO ID	*p*-value	Average FC*
Chlorophyll biosynthetic process	GO:0015995	0.000195	−1.6598
chlorophyll metabolic process	GO:0015994	0.000548	−1.1459
Photosynthetic electron transport in photosystem I	GO:0009773	5.77E-08	−1.3109
Photosynthetic electron transport in photosystem II	GO:0009772	0.000129	−1.4662
Electron transport chain	GO:0022900	9.89E-08	1.2090
Leaf development	GO:0048366	0.006600	1.1968
Phyllome development	GO:0048827	0.001743	−1.4103
Meristem development	GO:0048507	0.000969	1.6703
Meristem maintenance	GO:0010073	0.002897	1.4769
Regulation of meristem development	GO:0048509	0.001992	1.419
Phloem or xylem histogenesis	GO:0010087	0.000156	1.1578
Xylem development	GO:0010089	0.012376	2.0586
Lignin catabolic process	GO:0046274	0.015023	−3.0332
Lignin metabolic process	GO:0009808	0.000747	2.1952
Auxin metabolic process	GO:0009850	0.000685	1.6071
Tryptophan catabolic process	GO:0006569	0.009640	3.3018
Gibberellin metabolic process	GO:0009685	0.003229	4.7556
Gibberellin biosynthetic process	GO:0009686	0.015585	1.5146
*FC represents an average expression fold change of all DEGs that are involved in a biological process represented by a gene ontology.			

With the Pop’s pipe tool, we also performed protein domain enrichment analysis using the same set of DEGs as the input used for GO enrichment analysis. The significantly enriched protein domains are listed in Table 2. The proteins with the IPR003754 domain were presumably involved in chlorophyll biosynthesis given that tetrapyrroles^[46]^ and uroporphyrinogen III^[47]^ are large macrocyclic compounds for the biosynthesis of chlorophyll. The cellulose synthase (CesA) domain (IPR005150) was also significantly enriched in the DEGs; the changes of protein with this domain could be indirectly associated with the increased lignin content in some vascular bundles and vascular bundle sheaths as the biosynthesis pathways of lignin and cellulose are interconnected^[48,49]^. LIPOXYGENASE2 domain was also enriched in DEGs and was indicated to be involved in the biosynthesis of the oxylipin jasmonic acid^[12]^.

**Table 2:** Protein domains that were enriched in DEGs in *MIR319a* over-expression transgenics.

Domain	Description	No. of DEGs	EnrichScore	Average FC*
IPR008543	Chloroplast Ycf2	2	0.000295	−3.8012
IPR001344	Chlorophyll A-B binding protein	17	2.27E-16	1.0013
IPR002628	Photosystem II manganese-stabilizing protein PsbO	1	0.005071	1.0415
IPR003375	Photosystem I reaction centre subunit IV/PsaE	2	2.64E-06	1.0408
IPR003685	Photosystem I protein PsaD	2	1.05E-05	1.0476
IPR009806	Photosystem II protein PsbW, class 2	2	2.59E-05	−1.028
IPR001056	Photosystem II phosphoprotein PsbH	3	1.23E-06	−2.2731
IPR003754	Tetrapyrrole biosynthesis, uroporphyrinogen III synthase	1	0.0001913	−1.0524
IPR005150	Cellulose synthase	9	1.38E-06	1.5418
IPR001246	Lipoxygenase, plant	3	0.000962	1.1511
*FC represents average expression fold changes of all DEGs whose protein sequences have a specific protein domain.				

### Construction of miR319a regulatory gene network

**Figure 5:**
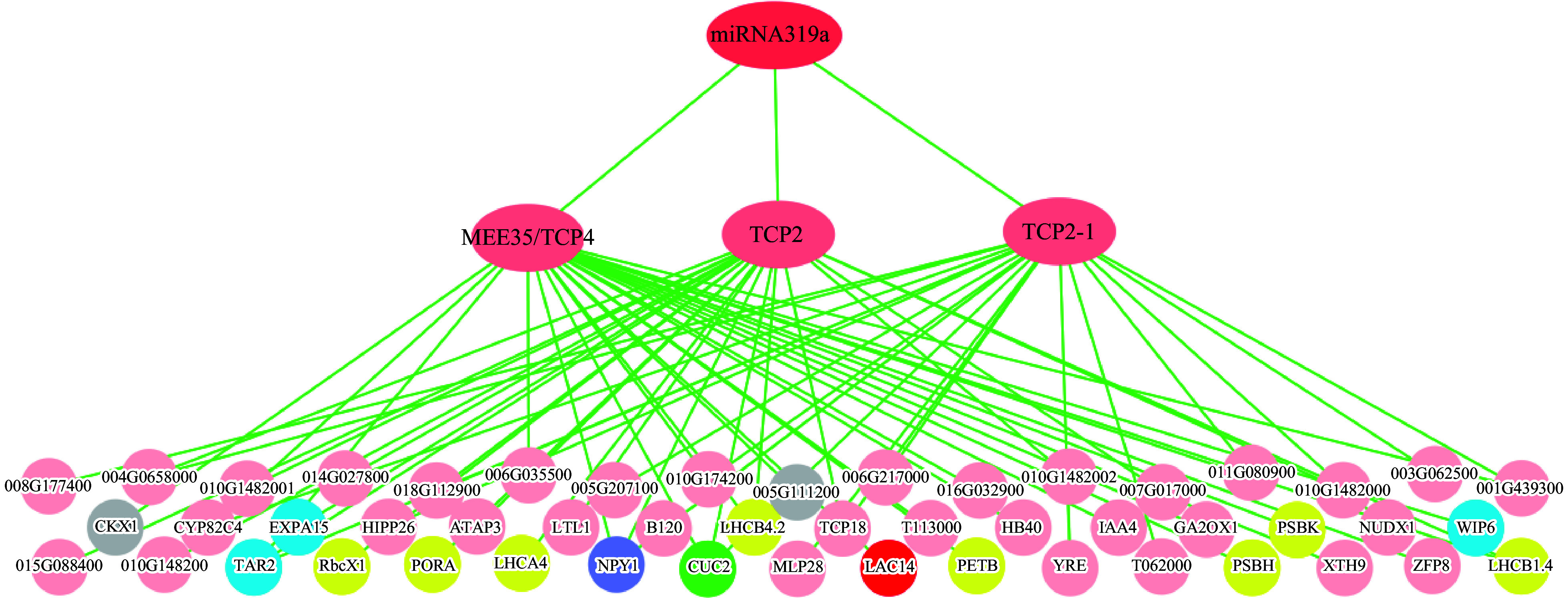
Three-layered hierarchical gene regulatory network under the control of miR319a in the leaves of *P. alba* × *P. glandulosa*. The green circle represents the leaf development gene, blue circles represent phloem or xylem histogenesis genes, the purple circle represents the phyllome development gene, gray circles represent xylem development genes, the red circle represents the lignin biosynthesis gene, and yellow circles represent photosynthesis genes. The gene IDs are provided in Supplemental Table 5.

As aforementioned, only three TCP genes, *MEE35/TCP4*, *TCP2* and *TCP2-1*, in 93 DEGs whose transcripts were targeted by miR319a, were differentially expressed in the *MIR319a* over-expression poplar transgenic lines, indicating that miR319a targets very small numbers of *TCP* genes for direct regulation in the leaves of over-expression transgenics. All the counterparts of the three genes in *A. thaliana* had recently proven to be the true target genes of At-miR319a^[45]^. In comparison to those in WT, all three genes were inversely down-regulated in *MIR319a* over-expression transgenics, supporting that these were direct targets of miR319a. We then used a top-down GGM algorithm^[32,33]^ to construct the gene regulatory network mediated by miR319a. The three genes, *MEE35/TCP4*, *TCP2* and *TCP2-1,* which had near-perfect complementarity in sequence with miR319a, were the direct target genes of miR319a (Fig. 5). The remaining DEGs in *MIR319a* over-expression transgenics were used as an input for inferring the third layer using a top-down GGM algorithm. We obtained many genes that function in leaf/phyllome development, and photosynthesis. For example, CUC2 is reported to mediate PIN1 convergence points and auxin maxima along the leaf margin^[50]^ , *NPY1* is highly expressed in leaf primordia and the double mutant line of *npy1* and *pid* change the phyllotaxis of leaf formation^[51]^. In addition, *TAR2*, *EXPA15*, and *WIP6* are involved in phloem or xylem histogenesis. As reported, *TAR2*, which encodes a trytophan aminotransferase, is an auxin biosynthetic gene required for HD-ZIP III-mediated xylem pattern^[52]^ and the WIP6 gene is implicated in regulating vein patterning^[53]^. *LAC14* is considered to be involved in polymerization of phenyl propanoid units and over-expression promoted lignification in poplars and reduced the proportion of syringyl/guaiacyl^[54]^. *LHCB1.4*, *LHCB4.2*, *LHCB4*, *PSBH*, *PETB*, *PSBK*, *PORA*, and *RbcX1* are involved in photosynthesis. *GA2OX1* is involved in the inactivation pathway of gibberellin^[55]^. In our prediction (Fig. 5), some bottom-layered genes, which were commonly regulated by two or three TCP genes in the middle layer, may be further characterized in future studies. Therefore, *miR319a* appears to control lignin biosynthesis, leaf development and differentiation, as well as photosynthesis, via *miR319-MEE35/TCP4*, *miR319*-*TCP2* and *miR319*-*TCP2-1* regulatory modules.

## DISCUSSION

We characterized the potential functions of miR319a in woody species poplar via a transgenic approach. The results showed that over-expression of miR319a had a great impact on leaf development and morphogenesis. The leaf curvature of *MIR319a* over-expression transgenics become wider and more jagged, which resemble the phenotypic changes observed in other miR319 transgenic lines of other species, including Pvi-miR319a in rice^[26]^, and Sly-miR319 in tomato^[24]^ (Fig. 1a). The termination of cell division at leaf edges between two lateral veins of *MIR319a* over-expression transgenics, resulted in visual jagged serration^[56-58]^, indicating that fewer hormones were generated and/or the diffusion of hormones to the areas between two lateral leaf veins became impeded. Such a phenomenon was also observed in *A.* *thaliana*^[11]^, tomato^[24]^, and rice^[26]^ upon miR319a over-expression.

We also observed dwarf phenotypes of *MIR319a* over-expression transgenic plants, indicating that overall growth and development are arrested when miR319a is ubiquitously expressed under the control of Cauliflower mosaic virus (CaMV) 35S promoter. Our study indicates that miR319a caused these phenotypic changes in leaves by directly regulating three *TCP* genes, *MEE35/TCP4*, *TCP2* and *TCP2-1*, which have near-complementarity with miR319a in sequences and were down-regulated significantly in response to miR319a over-expression (Supplemental Fig. 2). The previous studies in other species have revealed that TCPs can repress marginal meristem activity, thus promoting a switch from cell proliferation to cell differentiation^[59]^. In *A. thaliana*, miR319-TCP4-ARR16 module controls *de novo* shoot regeneration by affecting cytokinin responses^[60]^, and over-expression of each *Bra-MIR319* family member in *A. thaliana* inhibits cell division^[28]^.

We constructed a three-layered gene regulatory network which suggests that three TCP target genes may regulate genes involved in hormone metabolism-/transport-, photosynthesis-, and development-related processes in leaves, meristem and xylem. These results are largely in agreement with previous studies. For example, miR319a regulates *PvPCF5* whose over-expression affected leaf morphogenesis and cell proliferation in switchgrass^[27]^. In addition, indeterminate cell proliferation in leaf margins in *A. thaliana tcp* mutants has been linked with prolonged activity of *WOX*, *PRS* and *CYCB* genes^[45]^. Recent studies also showed that the miR319a-TCP module regulates miR396, a regulator of Growth-Regulating Factors (GRFs)^[61]^. The TCPs have also been shown to control auxin biosynthesis genes^[62]^, GA response^[63]^ and jasmonate biosynthesis^[12]^. Also, the *tcp* mutants show altered expression patterns for many photosynthesis-related genes in the different leaf domains, resulting in delayed differentiation, especially in the leaf margin area^[45]^. Among the 93 putative target genes with sequences complementary to miR319 in the poplar genome, two genes, *MYB65* and *ASA1*, have been implicated as being involved in hormone signalling. *ASA1* is involved in auxin biosynthesis and transport^[64]^ and *MYB65* is inducible by GA^[40]^. Phylogenetic analysis was performed to find evolutionary distances among three *TCP* genes *MEE35/TCP4*, *TCP2* and *TCP2-1*. Based on the phylogenetic tree obtained, we propose that *MEE35/TCP4* may have conserved functions with *AtTCP3*, *AtTCP4*, *AtTCP10* and *OsaPCF1*, and *TCP2* and *TCP2-1* may have the similar functions with *AtTCP2*.

In addition to its primary function in regulating leaf development and morphogenesis, miR319a had profound effects on many other biological processes. Gene ontology analysis of DEGs showed that up-regulation of miR319a affected the genes involved in chlorophyll synthesis, photosynthesis, leaf and phyllode development, meristem development and maintenance, regulation of meristem development, xylem histogenesis and development, lignin metabolism processes and hormone biosynthesis (Table 1). Protein domain enrichment analyses of DEGs revealed that protein families related to chlorophyll synthesis, photosynthesis and lipoxygenase biosynthesis were enriched (Table 2). As mentioned earlier, TCP4 activates the lipoxygenase 2 domain protein (*LOX2*) that catalyses the first step in the biosynthesis of oxylipin jasmonic acids^[12]^. Multicopper oxidoreductase laccase proteins have been shown to play a role in the polymerization of monolignols during lignin biosynthesis^[65]^. TCP4 can activate *VND7*, which is an upstream master regulator of secondary cell wall biosynthesis^[66]^. Over-expression of miR319a led to downregulation of its target TCPs, which inhibit the function of VND7 in secondary cell wall formation.

In *MIR319a* over-expression transgenic plants, we observed that more layers of cells in vascular bundles and vascular bundle sheaths were stained by phloroglucinol HCl, suggesting that more cells are lignified. However, the cells in vascular bundles of transgenics were stained dark red, while these in vascular bundle sheath cells were light red. These results indicate that lignin composition is likely to not be the same between vascular bundles and vascular bundle sheaths, and this needs additional research.

## CONCLUSIONS

Over-expression of miR319a in *P. alba × P. glandulosa* caused dwarf stature, narrow leaf blades, serrated leaf margins, and a high degree of lignification in some specific cells of the vascular system. The target gene analysis of miR319a, RNA-seq analysis of miR319 over-expression lines, and the construction of miR319a-mediated three-layered gene regulatory network together suggest that miR319-MEE35/TCP4, miR319-TCP2 and miR319-TCP2-1 were the three major regulatory modules controlling photosynthesis, hormone synthesis/metabolism/transport, and leaf development and differentiation related processes in *P. alba* × *P. glandulosa*, indicating the conserved functions of this ancient miRNA.

## MATERIALS AND METHODS

### Plant material and growth conditions

We obtained poplar plants (*P. alba* × *P. glandulosa*) from the tissue culture lab of the Chinese Academy of Forestry, Beijing, China. They were vegetatively propagated in Murashige and Skoog (MS) medium (pH 5.8) supplemented with 0.1mg/ml IBA and 1mg/ml NAA. The growth conditions included 16 h light/8 h dark cycle (light intensity ~300 μE m^−2^ s^−1^) at 25−28 °C. *MiR319a* over-expression transgenics and WT plants were grown in MiRacle-Gro® and Metro-Mix 200 soil mixture^[67]^.

### Generation of miR319a overexpression transgenic poplar

The plasmid *p35S-Osa-miR319a/p35S-hyg* for *MIR319a* over-expression was kindly provided by Prof. Dayong Li (Beijing Academy of Agriculture and Forestry Sciences). The plasmid was transformed into a *Agrobacterium tumefaciens* strain GV3101 by electroporation method, and the *Agroba*cterium-mediated transformation of *P. alba* × *P. glandulosa* was conducted as described previously^[68]^.

### Identification of miR319a targets from *P. trichocarpa*

The target genes were identified from *P. trichocarpa* transcripts using psRNATarget tool (http://plantgrn.noble.org/psRNATarget/) that can identify target DNA sequences based on complementary and energy levels of RNA: DNA duplexes as well as target site accessibility. The transcripts and annotation stored in this tool are the version 3.1 release.

### Phylogenetic analysis of miR319a target genes and *TCP* genes in poplar

All TCP protein sequences were retrieved from the UniProt database (https://www.uniprot.org). BLASTP of the TCP protein sequences against *P. trichocarpa* (https://phytozome.jgi.doe.gov) and *A. thaliana* (https://www.arabidopsis.org) protein sequences was undertaken to identify the homologous TCP proteins of poplar and *A. thaliana*. Multiple alignment of 93 poplar proteins (including three TCP proteins), five *A. thaliana* and two rice TCP proteins were constructed using Clustal X^[69]^. Using alignment file as an input, we then constructed an unrooted phylogenetic tree using MEGA7.0 with the following parameters: Neighbor-Joining method (NJ) with 500 bootstrap replicates, Minimal Evolution (ME) and Maximum Parsimony (MP), and expectation value ≥ 3.

### Anatomic analysis of leaf structures of miR319a over-expressed transgenics

We employed the phloroglucinol hydrochloric acid (HCl) staining method to stain lignin^[70,71]^. The leaf cross-sections of *MIR319a* over-expression transgenic plants and WT plants were excised carefully and preserved in a solution of ethanol (46%, v/v) and glacial acetic acid (46%, v/v). The sections were dipped in 1% (v/v) phloroglucinol HCl for 3 minutes, subsequently dipped in 92% ethanol, and then transferred into 25% HCl until they were stained. When some parts of the cross-sections turned red, the cross sections were shifted onto glass slides and mounted in a drop of fixative (glycerol, 25% lactic acid, 25% HCl, and phloroglucinol ethanol in the ratio of 50:40:7:3). The sections were observed under an optical microscope (Olympus, bx51).

### qRT-PCR analysis of miR319a expression in transgenic plants

We selected an average tree from each of the 14 *MIR319a* over-expression transgenic lines and three WT plants, collected young leaves and then immediately placed them into liquid nitrogen. Total RNAs were extracted using the standard CTAB method^[72]^ and gDNA was removed by DNase (Promega, M6101). Two µg of total RNAs were reverse transcribed to cDNA using the miRNA cDNA synthesis kit, with Poly (A) polymerase Tailing (Abm®). The qRT-PCR of mature miR319a was conducted as previously described^[73]^, with the primers TTGGACTGAAGGGTGCTCCC and ACGTCTGCCTGGGTGTCACGC.

### Quantification of gene expression levels in the leaves of *MIR319a* over-expression transgenic poplars using RNA-seq

Total RNAs were extracted using the standard CTAB method^[74]^ from the leaves of 14 transgenic lines and three WT plants for high throughput RNA library construction. The libraries were sequenced on an Illumina Hiseq 4000 sequencer using paired-end sequencing with the sequencing length equal to 150bp (Novogene, Beijing). RNA-seq reads were aligned with *P. trichocarpa* genome using HiSAT2 and subsequently FPKM values of each annotated gene was quantified using Cufflinks^[75]^. RNA-seq data were analyzed by comparing the gene expression between 14 transgenic and WT lines.

### Identification of DEGs and the enriched gene ontologies and protein domains in DEGs

The GO and protein enrichment analyses were conducted using the Pop’s pipes tool (http://sys.bio.mtu.edu/)^[76]^ that harbors three pipelines: DEG pipeline, gene ontology (GO) enrichment pipeline and protein domain enrichment pipeline. The DEG pipeline uses the edgeR package^[77]^ from Bioconductor with the significant cut-off threshold set to FDR corrected *p*-value ≤ 0.05. Both GO and protein domain pipelines use hypergeometric distribution to calculate the FDR *p*-value of each GO-term or domain.

### Construction of multilayered gene regulatory networks mediated by miR319a

We used a psRNATarget tool to identify the direct target genes of miR319a in the DEGs acquired from miR319a over-expression transgenic lines in comparison with a control. Three genes, *MEE35/TCP4* (Potri.001G375800), *TCP2* (Potri.004G065800) and *TCP2-1* (Potri.011G083100), were identified to directly target genes of miR319a. We then employed Top-down GGM algorithm to identify the target genes of these three *TCP* genes (namely miR319a’s indirect target genes)^[33]^ using Top-down GGM algorithm with the RNA-seq data of the rest of DEGs as input data.

## SUPPLEMENTARY DATA

Supplementary data to this article can be found online.
