# Supplementary figures and images for: MicroRNA319-mediated gene regulatory network impacts leaf development and morphogenesis in poplar
Source: For Res (Fayettev). 2021 Feb 5;1:4. doi: 10.48130/FR-2021-0004 (PMC11524277; doi:10.48130/FR-2021-0004)

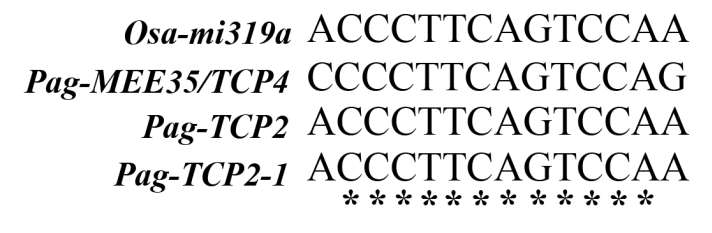

Supplement: Supplementary file 1 — Supplementary data to this article can be found online. [file FR-2021-0004-S1.zip › 10.48130_FR-2021-0004-Suppl-FigureS2.png]

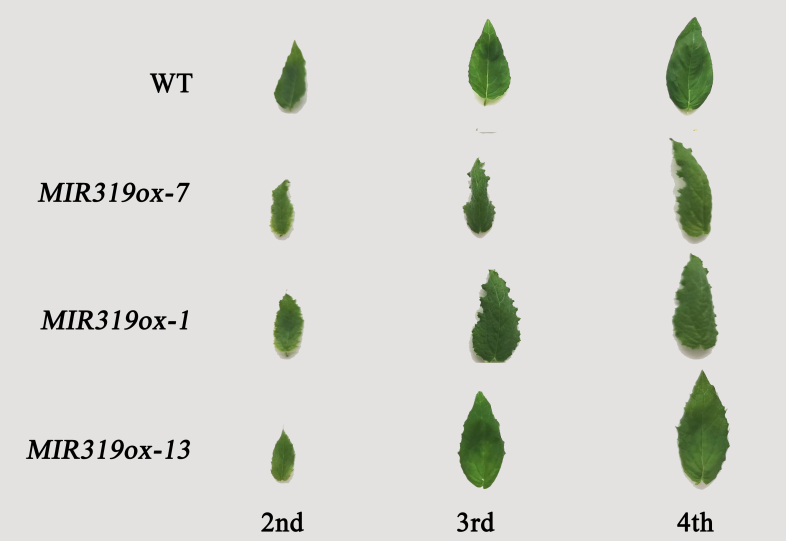

Supplement: Supplementary file 1 — Supplementary data to this article can be found online. [file FR-2021-0004-S1.zip › 10.48130_FR-2021-0004-Suppl-FigureS1.png]
